# PAH exposure and associated health risks can be high at a fire site both during fire and long after the fire is extinguished

**DOI:** 10.1093/annweh/wxag003

**Published:** 2026-02-04

**Authors:** Bo Strandberg, Karin Lovén, Vilhelm Malmborg, Jennie Özdemir, Joakim Pagels, Maria Hedmer, Lina Hagvall

**Affiliations:** Division of Occupational and Environmental Medicine, Department of Laboratory Medicine, Lund University, Scheelev. 2, 221 00 Lund, Sweden; Department of Occupational and Environmental Medicine, Skåne University Hospital, Scheelev. 8, 223 81 Lund, Sweden; Division of Occupational and Environmental Medicine, Department of Laboratory Medicine, Lund University, Scheelev. 2, 221 00 Lund, Sweden; Department of Occupational and Environmental Medicine, Skåne University Hospital, Scheelev. 8, 223 81 Lund, Sweden; Ergonomics and Aerosol Technology, Department of Design Sciences, LTH, Lund University, Box 118, 221 00 Lund, Sweden; Department of Occupational and Environmental Medicine, Skåne University Hospital, Scheelev. 8, 223 81 Lund, Sweden; Ergonomics and Aerosol Technology, Department of Design Sciences, LTH, Lund University, Box 118, 221 00 Lund, Sweden; Division of Occupational and Environmental Medicine, Department of Laboratory Medicine, Lund University, Scheelev. 2, 221 00 Lund, Sweden; Department of Occupational and Environmental Medicine, Skåne University Hospital, Scheelev. 8, 223 81 Lund, Sweden; Division of Occupational and Environmental Medicine, Department of Laboratory Medicine, Lund University, Scheelev. 2, 221 00 Lund, Sweden; Department of Occupational and Environmental Medicine, Skåne University Hospital, Scheelev. 8, 223 81 Lund, Sweden

**Keywords:** PAH, PAC, firefighter, forensic technician, occupational exposure, passive samplers PUF

## Abstract

The International Agency for Research on Cancer has recently classified occupational exposure as a firefighter as carcinogenic (group 1A). Even though the occupation of firefighter is associated with high exposure to pollutants, it is challenging to carry out exposure studies to assess health risks due to the extreme conditions associated with firefighting. Routine monitoring of occupational exposure to polycyclic aromatic hydrocarbons (PAHs) via active sampling of air is thus not feasible; however, polyurethane foam (PUF) passive air samplers are robust enough to enable monitoring of PAH in occupations such as firefighting. Two measurement campaigns were carried out at a firefighter training facility in Sweden. In the first, PAH concentrations in air were measured for firefighters, observers, and post-fire workers during fire extinguishing exercises. A wide range of PAH exposures were found; firefighters' exposures were highest (8,300 to 760,000 ng m^−3^), followed by those of observers (1,600 to 11,000 ng m^−3^) and post-fire workers (120 to 3,600 ng m^−3^). In the second, PAH concentrations in air were measured inside 2 burned out sheds for 38 d, starting 3 h after the end of the fire extinguishing exercise. Gas-phase PAH concentrations inside the shed after the fire was extinguished subsided rapidly initially but were high even a week after the fire (3,000 ng m^−3^) and were 15 to 20 times higher than ambient air levels after more than a month. Mechanical agitation or stirring ashes during post-firework may lead to elevated exposure to the more carcinogenic PAHs, which have higher molecular weights. The results indicate that PAH exposure can be high at a fire site both during the fire and for weeks after the fire is extinguished. Simple preventive measures such as postponing investigation of a fire site for at least a week and wearing respiratory protection can decrease occupational exposure to PAHs.

What's Important About This Paper? This study reports airborne exposures and concentrations of 35 PAH compounds and 6 dibenzothiophene derivatives during firefighting training activities and post-fire. Even weeks after a fire, the levels of PAH can be noticeably elevated in indoor fire sites. The study also demonstrates the utility of a passive polyurethane foam short-term personal sampler for this application.

## Introduction

Firefighting is a high-risk occupation exposing firefighters to both immediate danger and carcinogenic pollutants. In 2023, the International Agency for Research on Cancer classified occupational exposure as a firefighter as carcinogenic to humans (Group 1) (Demers et al. 2022). Firefighters are exposed not only to carcinogenic pollutants from the smoke but also to other carcinogens like shift work or asbestos. There is further a risk of secondary exposure to pollutants before or after extinguishing operations from contaminated material, protective clothing, or other equipment (Baxter et al. 2014; Oliveira et al. 2020). Examples of places where secondary exposure can occur are vehicles, areas for material care, changing rooms, and common rooms. Studies have shown that secondary exposure can constitute a large part of the total exposure to gases and particles in firefighting exercises and accidental fires (Baxter et al. 2014; Rogula-Kozlowska et al. 2020; Lovén et al. 2024).

Polycyclic aromatic hydrocarbons (PAHs) are key pollutants in fire smoke from a toxicological perspective. PAHs are semi-volatile compounds, distributed between the gas and particle phases in air depending upon the number of rings in the molecular structure. Typically, PAH toxicity is evaluated for the 16 PAHs prioritized by the United States Environmental Protection Agency (EPA), of which benzo[a]pyrene (BaP) is considered to be the most carcinogenic (Zelinkova and Wenzl 2015). The toxicity of a measured exposure to a mixture of PAH can be estimated by performing benzo[a]pyrene equivalence (BaP_eq_) calculations using toxic equivalency factors (TEFs). However, calculations of PAH exposure toxicity using benzo[a]pyrene equivalents in work environments are sparse (Kirk and Logan 2015; Oliveira et al. 2017; Rogula-Kozlowska et al. 2020; Strandberg et al. 2022; Kander et al. 2024; Kander et al. 2025). In addition, there are indications that compounds other than the known carcinogenic native PAHs, eg alkylated PAHs and dibenzothiophenes (DBTs), contribute to the toxicity of the smoke (Wobst et al. 1999; van Rooij and Jongeneelen 2007; Poutasse et al. 2020) and are relevant to include in smoke PAH exposure assessment.

Measuring firefighters' personal exposure to air pollution during extinguishing operations can be a difficult task because of their challenging and dangerous work environment. An active sampling technique can be obtrusive and logistically difficult or even unusable under firefighting conditions (Fent et al. 2014; Wingfors et al. 2018). Alternative ways to measure PAH exposure are by using portable passive air samplers (PAS) in the form of polyurethane foam (PUF-PAS) or silicon materials shaped as wristbands or tags (Bohlin et al. 2010a, 2010b; Strandberg et al. 2018; Poutasse et al. 2020; Gren et al. 2022; Strandberg et al. 2022; Bonner et al. 2023; Keir et al. 2023). The PUF-PAS sampler has been calibrated for uptake rates for both gaseous and particulate-associated PAH compounds in short exposure times (2 to 8 h) among firefighters and forensic technicians (Strandberg et al. 2018). However, PUF-PAS has previously not been used for sampling of both gas and particle phase PAH during the challenging sampling conditions of fire extinguishing. It is important to obtain a quantitative measure of both gaseous and particulate PAH exposure during firefighting work, as exposures can occur through inhalation and dermal contact, including as a result of penetration of protective clothing (Wingfors et al. 2018; Bonner et al. 2023). Detailed exposure data on PAHs and derivatives such as alkylated PAHs or DBTs for firefighters and post-fire workers or risk assessment of the exposures has not been reported during a full work shift. Furthermore, investigations of the decline of indoor air levels of PAH, alkylated derivatives, or DBT in the aftermath of a fire has previously not been published.

Therefore, the aims of this study were to:

1. Quantify personal air exposure levels and sources of 35 PAHs and 6 DBTs (parent compounds and alkylated species) and evaluate the PAH exposure toxicity as BaP equivalents for firefighters, observers, and post-fire workers during a full work shift.
2. Quantify post-fire air concentrations of PAH and DBT as a pilot study in 2 enclosed burned-out sheds over 38 d and assess the associated risks for post-fire workers.

## Material and methods

### Environmental settings

The first part of the study (referred to as part 1) focused on personal exposure measurements and was performed during real fire extinguishing exercises during October and November 2021 at a firefighter training school in Sweden, where firefighters and forensic investigators are educated. Students were fitted for their self-contained breathing apparatus (SCBA) and protective clothing onsite. At the school, a team of permanent staff handles preparation before and cleaning/dismantling after the fire extinguishing exercises. Personal exposure measurements were performed on 4 d during different exercises. These have previously been described (Lovén et al. 2024), and a brief description is given here. Fire extinguishing exercises were performed during days 1 to 3. The exercise on day 1 was a standard fire extinguishing exercise and used only wood fuel and fire initiator. The exercises during days 2 to 3 were performed in 8 sheds consisting of 1 or 2 rooms (4 of each) furnished individually as both residences and workplaces. During day 4, personal measurements for post fire workers were performed. These workers carried out service and material maintenance such as cleaning out standard exercise training buildings and reloading with new fuel or cleaning and maintenance of personal protective equipment and firefighting equipment (radio, flashlight, etc.). The average outdoor temperature for all 4 d was 13 °C.

The second part of the study (referred to as part 2) focused on determining post fire air concentrations over time and was performed inside two of the sheds after the fire extinguishing exercise, when the burned-out sheds were closed. The sheds had no electricity, plumbing, or mechanical ventilation. The only ventilation consisted of draught through the broken windows covered with shutters and cracks in the building envelope. The sheds were left undisturbed during the measurement period (38 d) except for 1 to 2 occasions when forensic investigations were performed for educational purposes. It is not known when these exercises took place. The outdoor temperature during the second part of the study ranged between −1.1 and 14.2 °C with a mean temperature of 7.5 °C (Knivsåsen meteorological station; SMHI 2024). The temperature inside the sheds was not measured but assumed to be the same as the outdoor temperature, as there was no heating in the sheds.

### Part 1: Personal air exposure to PAH and DBT

Personal air exposure measurements of PAHs and DBTs using PUF-PAS were performed for 23 participants, including: firefighters (*n* = 13), observers (*n* = 4), and post-fire workers (*n* = 6). The observers included policemen (*n* = 2) and other personnel positioned around the fire scene (*n* = 2). The post-fire workers included forensic technicians (*n* = 2) as well as technical and service personnel (*n* = 4). For details on the participants, see Lovén et al. (2024). In brief, the firefighters wore protective clothing and SCBAs during the fire extinguishing exercises. Observers, post-fire and the technical and service personnel wore work clothes. The forensic technicians wore coveralls and full-face masks with P3 filters when entering the sheds after the fire was extinguished.

The firefighters were positioned outside the buildings or sheds when the fire started. All firefighters observed the exercise at 10 to 40 m from the fire and donned SCBAs during the extinguishing exercise they themselves participated in. The exercises had durations of 5 to 30 min. During the exercise, observers moved around the fire scene approximately 20 m from the fire but sometimes moved to within a few meters to document the extinguishing process, thus moving in and out of the smoke plume. The air sampling was performed either over a full day (5.5 to 8 h) or half a day (2 to 3.5 h). The participants were usually dressed in their protective clothing or coveralls throughout the work shift.

In addition, stationary air sampling was performed approximately 20 m from the fire scenes. Ambient air sampling was performed approximately 200 m from the fire scene.

### Part 2: Post fire PAH and DBT air concentrations

PAH concentrations were measured using PUF-PAS. Two of 8 sheds were selected: One was furnished as a living room with TV and bedroom (Shed 1: 2 rooms, volume 41 m^3^) and the other as a bedroom with TV (Shed 2: 1 room, volume 20.5 m^3^). Simulated causes of fire were electrical malfunction in a vacuum cleaner (Shed 1) and arson in the form of petrol and lighted match distributed from the open window (Shed 2). Air sampling was performed using 7 sequential measurement periods of increasing length (Table S1). The first measurement started about 3 h after the fire was extinguished and lasted until the next day, the second period lasted 2 d, and the following 5 measurement periods lasted 1 wk up to 38 d after the fire. Stationary sampling of ambient air was also performed at the sampling place 200 m from the sheds over 2 sequential periods of 2.5 wk to avoid volatilization or degradation of naphthalene (Harner et al. 2013).

### Air sampling methods

For the personal sampling, a cylindrical polyurethane foam passive air sampler was used (PUF-cyl, length: 10 cm, diameter: 2.2 cm, total surface area: 77 cm^2^: density: 0.030 g cm^−3^, Klaus Ziemer GmbH, Germany) and placed inside a protective cover net (diameter: 2.2 cm, length: 10 cm, mesh size: 1.0 mm, AB Derma, Sweden). The sampler was mounted in the personal breathing zone, clipped to the hood of the firefighter turnout gear or to the clothing at the chest for observers and post-fire workers. For the outdoor stationary measurements, the PUF-cyl was housed in stainless steel domes (Tisch Environmental, Inc., OH, USA). The domes were semi-open to prevent gravitational deposition of coarse particles and sheltered from rain but were open to air on all sides, thus mimicking the personal measurements. These samplers were mounted at a height of approximately 1.5 m, corresponding to the personal breathing zone. This study was part of a larger project involving biomonitoring of PAH exposure in all occupational groups studied (Lovén et al. 2024). The project was approved by the Swedish Ethical Review Authority, Sweden (registration no. 2021–04138) and performed in accordance with the Declaration of Helsinki, including obtaining informed written consent from all participants.

In part 2 of the study, PUF-cyl samplers were placed inside the burned-out sheds at a height of approx. 1 to 1.5 m and were housed in a stainless steel dome on a tripod support (Wilford et al. 2004; Bohlin et al. 2008). For the stationary ambient air sampling 200 m away from the shed, the traditional polyurethane foam disc sampler design (Harner et al. 2013; Bohlin et al. 2014a, 2014b; Parnis et al. 2016) was used (14 cm in diameter, 1.2 cm thickness, surface area 360 cm^2^, density 0.035 g cm^−3^ [Klaus Ziemer GmbH, Germany]) and housed in stainless steel domes (Tisch Environmental, Inc., OH, USA).

Previously published uptake rates for the PUF-cyl sampler for all PAHs in gaseous- and particulate phases, respectively, including alkylated species were used for the calculations of concentrations of PAHs (Strandberg et al. 2018). The DBT concentrations were calculated using uptake rates of closely related PAH compounds based on the assumption that they are quite the same (Harner et al. 2013). All results are time-integrated concentrations.

For the post-fire study (part 2), the uptake rates for PUF-cyl for parent and alkylated PAHs have been estimated from workplace measurements covering 2 wk using the same sampler and shelter as in this study (Bohlin et al. 2010a, 2010b), as uptake rates are slightly lower for longer sampling times. The PUF-disc sampler design used in the stationary outdoor ambient air measurement has been calibrated for uptake-rates covering 4 wk for both gaseous and particulate associated PAH compounds including alkylated species and DBTs (Harner et al. 2013; Bohlin et al. 2014a, 2014b; Parnis et al. 2016).

### Chemical analysis

Chemical analysis of the following compounds was performed: 19 parent PAHs including the 16 US EPA priority PAHs, benzo[e]pyrene, perylene, and coronene, as well as 16 alkylated PAHs and DBTs (dibenzothiophene and 5 alkylated dibenzothiophenes) (Table 1, Table S2). Herein, Sum 35 PAH refers to the sum of 35 PAHs (parent PAH compounds plus alkylated species), L-PAHs refers to low molecular weight PAHs (>90% in gaseous phase), M-PAHs refers to intermediate (medium) molecular weight PAHs (fractions in both gaseous and particulate phases), H-PAHs refers to particle bound, high molecular weight PAHs (>90% on particles).

**Table 1:** Air concentrations (ng m^−3^), geometric means (GMs) and ranges, determined via passive (PUF-cyl) sampling of PAH categories (L-, M- and H-PAHs), 16 US EPA PAHs, alkylated PAHs, Sum 35 PAH, and DBTs on firefighters and observers during controlled house burns and on post-fire workers performing service and material maintenance after the fire, and 2 stationary measurement locations, 20 and 200 m away, respectively.

	Firefighters		Observers		Post fire workers		Stationary 20 m away		Ambient air 200 m away	
	GM (*n* = 13)	Range	GM (*n* = 4)	Range	GM (*n* = 6)	Range	GM (*n* = 3)	Range	GM (*n* = 3)	Range
L-PAHs	90,000	6,300–580,000	4,100	1,600–10,000	480	91–3,400	11,000	4,000–27,000	79	30–330
L-PAHs (excl. Naphthalene)	26,000	1,800–190,000	520	190–2,000	77	16–450	4,600	1,300–14,000	34	15–120
M-PAHs	1,300	54–14,000	11	2.6–50	2.6	0.49–6.6	590	180–2,100	12	5.0–39
H-PAHs	3,600	170–41,000	21	3.9–110	3.6	0.78–27	82	17–330	2.9	1.6–4.3
16 US EPA PAHs	96,000	6,700–640,000	3,700	1,600–10,000	490	94–3,500	12,000	4,200–29,000	95	38–370
Alkylated PAHs	20,000	15,000–120,000	76	21–330	76	33–380	2,600	790–6,600	34	18–59
Sum 35 PAH	120,000	8,300–760,000	3,800	1,600–11,000	590	120–3,600	15,000	5,000–36,000	130	57–430
Total DBTs	32	1.3–390	0.25	0.058–1.5	ND	ND	6.4	1.5–27	1.2	0.69–3.4

The analytical procedures and instrumentation used are described in detail in the Supplementary Information (SI) and are given here in brief. The samples were spiked with a deuterated internal standard (IS) mixture containing the 16 US EPA PAHs. A Dionex ASE 350 Accelerated Solvent Extractor (Thermo Fisher Scientific, Inc., MA, USA) was used for extraction of the PUF-PAS samples. Extracts were then cleaned up through Pasteur pipettes filled with 2 cm of silica gel, solvent exchanged to n-hexane and then concentrated by purging with nitrogen to a final volume of 40 to 70 µL. Target compounds were separated on an Agilent 8890 GC System gas chromatograph coupled to an Agilent 7010B GC/TQ triple mass spectrometer (MS). Quantification of the compounds in the sample is based upon the IS method as described in Loive et al. (2024).

### Quality assurance/quality control

PUF-PAS blanks (PUF-cyl and PUF-disc) were analyzed in parallel with the samples. Some minor residues were found in the blanks, but in no case was the amount of any compound in any blank greater than 10% of the amount in any sample. All PAH results were blank-corrected. The limit of detection (LOD) was calculated as 3 times the standard deviation of the PAH and DBT compound in the blanks or the analytical background noise of those blanks.

Duplicate sampling using PUF-PAS was performed in 4 of the measurements (part 1), on one observer, 2 post-fire workers (Fig. S1) and, at the stationary sampling place 20 m away. Duplicate sampling of the firefighter group could not be performed for practical reasons. Duplicate measurements were performed for 5 of the 7 measurement periods in the post-fire study (part 2) and alternated between the 2 sheds (Fig. S2).

Two certified reference materials (SRM 1649a and 1649b urban dust) were used as quality control (QC) samples. The measured levels of PAHs rarely deviated more than 30% from the certified levels (Fig. S3). A more detailed discussion of the QC results is provided in the Supplementary Information.

### PAH exposure toxicity

To estimate the total toxicity of PAH mixtures in the studied occupational groups (part 1) and the post-fire study (part 2), equivalents of benzo[a]pyrene (BaP_eq_), were calculated by multiplying concentrations of the 16 US EPA PAHs with their respective toxic equivalent factor (TEF) value, with the TEF for BaP set to one (Nisbet and LaGoy 1992; Malcolm and Dobson 1994; Larsen and Larsen 1998; Lloyd and Denton 2005). The respective BaP_eq_ were added together for each exposure scenario to achieve a total BaP_eq_.

### Statistical analysis

Descriptive statistics for the exposure data is presented as geometric mean (GM) and as minimum (min) and maximum (max) values. Sample results < LOD were set to half of LOD. For the statistical analysis, IBM SPSS Statistic 28 software for Windows was used. Q–Q-plots indicated that the data were approximately log-normally distributed. Correlations between air levels of PAH and derivatives were analyzed using bivariate Spearman correlation analysis. The level of significance was set at *P* < 0.05.

## Results

### Part 1

Table 1 shows descriptive statistics for PAH and DBT compounds measured in part 1 of the study, and Table S2 shows results for the individual compounds. There were large differences in levels between the 3 occupational groups but also within each occupational group. The exposure to Sum 35PAH was highest for firefighters (120,000 ng m^−3^, range 8,300 to 760,000 ng m^−3^) followed by observers (3,800 ng m^−3^, range 1,600 to 11,000 ng m^−3^) and post-fire workers (590 ng m^−3^, range 120 to 3,600 ng m^−3^). Sum 35PAH concentrations 20 m downwind from the fire were also noticeably elevated (15,000 ng m^−3^), and concentrations 200 m from the fire was 130 ng m^−3^.

Seven L-PAH constituents accounted for more than 85% of the total Sum 35PAH in all samples. Of these compounds, naphthalene was the most dominant PAH and constituted more than 50% of most samples followed by acenaphthylene, biphenyl, 2-methylnaphthalene, 1-methylnaphthalene, phenanthrene, and fluorene. However, there is a difference in pattern between the occupational groups. For firefighters, L-PAHs made up approximately 94% of Sum 35PAH, compared to >99% among observers and 80 to 99% among post-fire workers. The carcinogenic H-PAHs constituted about 4% of the total Sum 35PAH for firefighters, but for observers, this percentage was insignificant (<0.5%). For 4 of the post-fire workers, the H-PAH percentage was insignificant (<0.5%), but for 2 others, it was high, 2% and 18%, respectively.

Alkylated species constituted 2 to 25% of the Sum 35PAH concentrations. All alkylated PAH species included in the analysis method except 5-methylchrysene were detected in the samples. For DBTs, the mean air levels were 32 ng m^−3^, 0.25 ng m^−3^ and below the LOD for firefighters, observers, and post-fire workers, respectively.

Among firefighters, there were strong correlations between personal exposure concentrations for L-PAHs, M-PAHs, H-PAHs, alkylated PAHs, and DBTs (Table 2), but this pattern was not seen in the exposure of post-fire workers, for whom strong correlations were only seen between exposures to L-PAH, alkylated PAH and to the 16 EPA prioritized PAH (Table 3). The sample size for the observer group was too small to perform the correlation analyses.

**Table 2:** Results from correlation analysis of air levels of PAH and DBT in the personal breathing zone of firefighters.

Exposure marker	16 US EPA PAH^a^	L-PAH	M-PAH	H-PAH	alk-PAH^b^	DBT^c^
16 US EPA PAH	—	1.0**	0.819***	0.775**	0.989***	0.890***
L-PAH		—	0.819***	0.775**	0.989***	0.890***
M-PAH			—	0.962***	0.802***	0.659*
H-PAH				—	0.775**	0.698**
alk-PAH					—	0.907***
DBT						—

Spearman's rank correlation coefficient (*r*_s_) is presented, and statistical significance noted with * for *P* < 0.05, ** for *P* = 0.01–0.001, and *** for *P* < 0.001.

^a^EPA prioritized 16 PAH; ^b^alk-PAH, alkylated PAH; ^c^DBT, dibenzothophenes.

**Table 3:** Results from correlation analysis of air levels of PAH and DBT in the personal breathing zone of post-fire workers.

Exposure marker	16 US EPA PAH^a^	L-PAH	M-PAH	H-PAH	alk-PAH^b^	DBT^c^
16 US EPA PAH	—	1.0**	0.77	0.14	0.94**	0.39
L-PAH		—	0.77	0.14	0.94**	0.39
M-PAH			—	0.60	0.71	0.13
H-PAH				—	0.09	−0.13
alk-PAH					—	0.66
DBT						—

Spearman's rank correlation coefficient (*r*_s_) is presented, and statistical significance noted with * for *P* < 0.05, ** for *P* = 0.01–0.001 and *** for *P* < 0.001.

^a^EPA prioritized 16 PAH; ^b^alk-PAH, alkylated PAH; ^c^DBT, dibenzothophenes.

### Part 2

**Figure 1:**
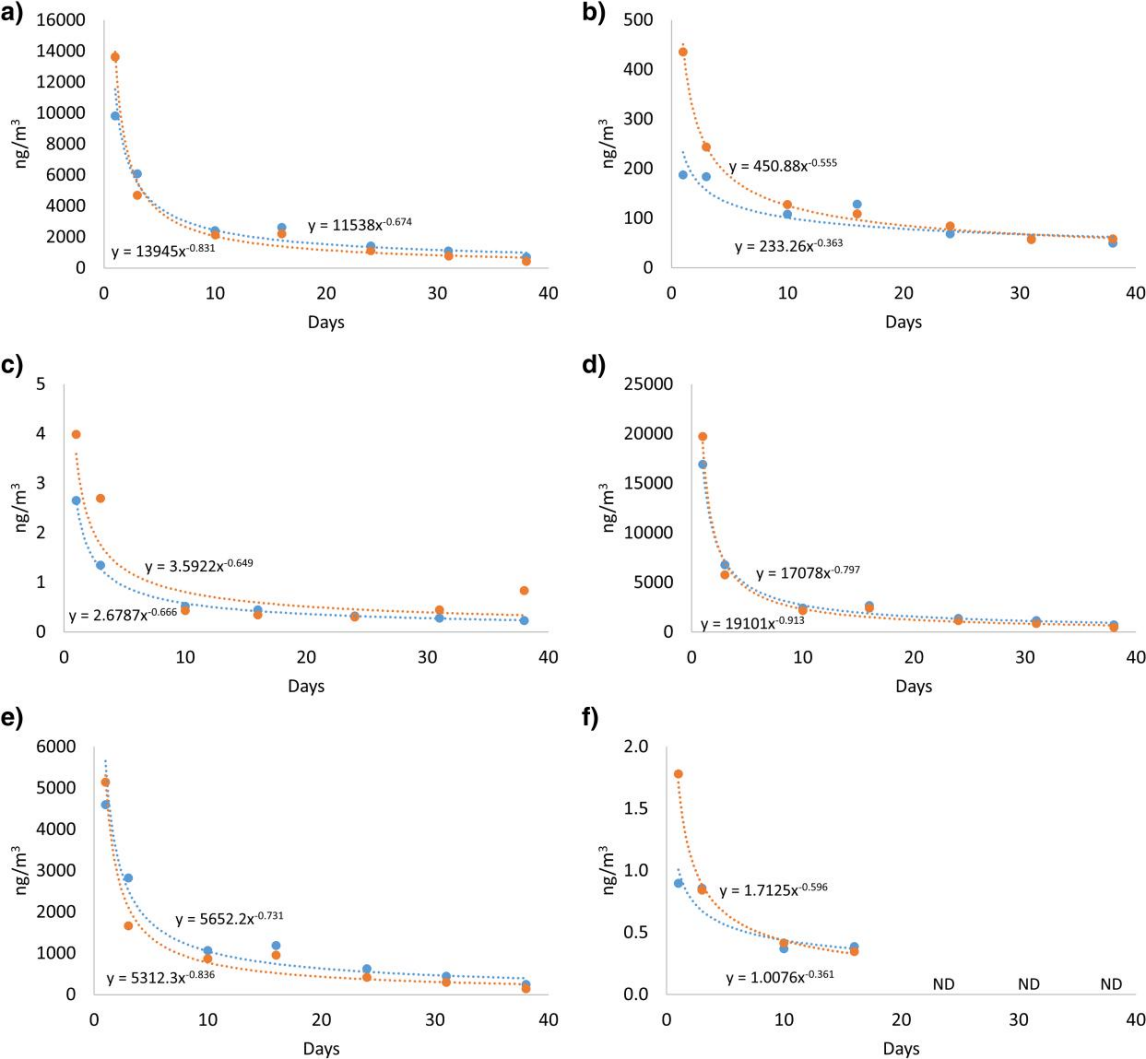
Air concentrations of (a) L-PAH excluded naphthalene, (b) M-PAH, (c) H-PAH, (d) US EPA 16 PAH, (e) alkylated PAH, and (f) dibenzothiophene in shed 1 (blue) and shed 2 (orange). ND, not detected.

Figure 1 and Table S1 show concentrations of Sum 35PAH and DBTs in 2 burnt-out sheds over 38 d. Results for individual compounds are presented in Table S3. The indoor levels of gas phase L-PAH were high during the first day after the fire (22,000 and 25,000 ng m^−3^) (Table S1). L-PAH levels in the sheds dominated the PAH pattern and accounted for >95% of the total Sum 35PAH, similarly to the fire-extinguishing exercises (part 1). Naphthalene was the most dominant PAH compound and constituted approximately 50% of the total Sum 35PAH level.

There were strong correlations between indoor air levels of L-PAH and M-PAH (*r*_s_ = 1.0, *P* < 0.01), M-PAH and H-PAH (*r*_s_ = 0.82, *P* = 0.023), and finally L-PAH and H-PAH (*r*_s_ = 0.82, *P* = 0.023) in shed 1. In shed 2, a strong correlation was seen between air levels of L-PAH and M-PAH (*r*_s_ = 0.93, *P* < 0.01). However, no statistically significant correlations between air levels of M-PAH and H-PAH or L-PAH and H-PAH were seen. Furthermore, there were strong correlations between indoor air levels of L-PAH, M-PAH and H-PAH between the sheds, respectively (Table S4) indicating a similar pattern of decline in levels of PAH and derivatives, despite the differences in volume and furnishing of the sheds as well as the differences in fire scenarios.

Stationary air measurements outside the sheds showed Sum 35PAH concentrations for 38 d after the fire were an order of magnitude lower than those measured during the 3 d the personal measurement campaign was carried out (part 1 of study), except for naphthalene where the concentrations were similar (Table 1). Levels were comparable to an urban environment during the summer in southern Sweden (Loive et al. 2024).

### Estimation of PAH exposure toxicity

**Figure 2:**
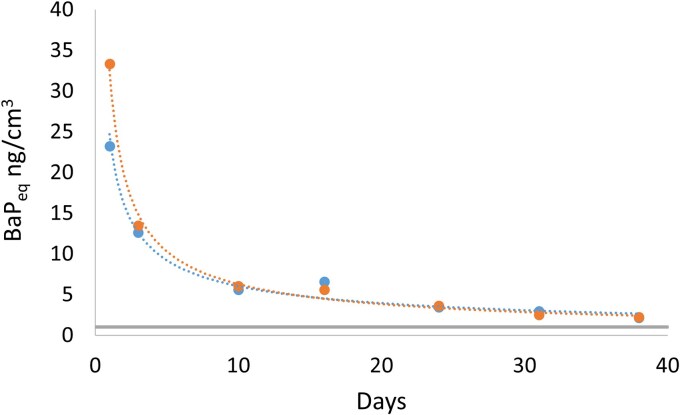
Estimation of PAH exposure toxicity for part 2 of the study (3 h after the fire was extinguished to 38 d) using toxic equivalence factors (TEF) of benzo[a]pyrene (BaP_eq_) of 16 parent PAHs for shed 1 (blue) and shed 2 (orange). The results of the measurement periods refer to the average level for the days shown. The gray line indicates the European Community annual BaP_eq_ standard on PM10 in ambient air of 1 ng m^−3^.

PAH toxicity estimates were calculated using BaP_eq_ to compare the toxicity of the Sum 35PAH exposure during different working tasks (Table 4). These values do not represent toxicity to participants as they do not account for exposure duration or the use of respiratory protection. Geometric means (GM) for Sum 35PAH exposure were 790 ng m^−3^ BaP_eq_ for firefighters, 7.9 ng m^−3^ BaP_eq_ for observers and 1.2 ng m^−3^ BaP_eq_ for post-fire workers. In study part 2, the toxicity of Sum 35PAH exposure based on measurements inside the sheds was found to be more than 20 ng m^−3^ BaP_eq_ the first day after the fire, and decreased according to a power law function to 2 ng m^−3^ after 38 d (Fig. 2). In the first measurement period, the L-PAH contributed the most to the total toxicity, however, with time M-PAH dominated (Table S5).

**Table 4:** Estimation of PAH toxicity for all individual exposures in firefighters, observers, and post-fire workers using BaP_eq_ and toxic equivalence factors (TEF) of the 16 EPA PAHs.

	BaP_eq_ GM, ng m^−3^	BaP_eq_ range (min–max), ng m^−3^
Firefighters	790	47–7,000
Observers	7.9	2.4–35
Post-fire workers	1.2	0.17–5.3

The samplers worn by the firefighters were mounted on the outside of their self-contained breathing apparatus.

## Discussion

This study expands upon previously reported data about exposures during fire extinguishing exercises at a training facility for firefighters (Lovén et al. 2024) by reporting data about personal and stationary measurements of the concentration of PAHs, alkylated derivatives, and DBT derivatives during a full work shift.

Concentrations of the 16 US EPA PAHs measured in this study in the breathing zone of firefighters, observers, and post-fire workers (Table 1, Table S2) were comparable to or lower than those measured in other firefighter exposure studies. High concentrations during firefighting exercises have been reported during controlled burns for firefighters; 5 to 20 mg m^−3^ (Fent et al. 2014) and 20 to 40 mg m^−3^ (Wingfors et al. 2018). These studies investigate PAH levels during the extinguishing work only and not, as in the present study, the levels as time integrated means over an entire work shift or working day. Concentrations of the 16 US EPA PAHs of 1,400 to 12,000 ng m^−3^ have previously been reported for firefighter team leaders standing 5 to 10 m from the entrance of the building and, 71 to 23,000 ng m^−3^ for forensic investigators, entering the site within 12 h to 5 d after the fire (Sjöström et al. 2019). These results are comparable to those of observers and post-fire workers in the present study.

The strong correlation between DBT concentrations and the concentration of the 16 US EPA PAHs among firefighters (Table 2) indicates that the fire event is the source of exposure to DBTs. There are few studies that have shown that smoke can be a source of DBTs (Wobst et al. 1999; Poutasse et al. 2020). However, the PAH subgroups correlated poorly for the post-fire worker group (Table 3), which may reflect that these workers have multiple, different sources of exposure with different PAH composition, such as secondary emissions from materials and equipment during maintenance of the fire equipment and personal protective equipment, and preparation and cleaning of exercise buildings before and after fire-extinguishing exercises. However, for 2 post-fire workers, the levels of H-PAH and of BaP, one of the most carcinogenic classes of PAH, were elevated, demonstrating that post-fire workers can be exposed to elevated levels of PAHs in both the gaseous and particulate phases.

The study observed that PAHs persist in indoor, burned areas, as the concentrations in burned sheds were still 15 to 20 times higher than outdoor concentrations 1 month after burning. For some individual PAHs in the gas phase, eg, phenanthrene and fluoranthene, the levels after over a month were almost 200 and 60 times higher than the outdoor ambient air levels, respectively (Tables S1 and S3). Concentrations and loss rates in the 2 separate sheds were similar for all studied compound groups, respectively, despite the buildings being differently furnished and having different total volumes. Total Sum 35PAH and DBT levels in both the gas and particle phases were seen to decrease according to power law functions over time in the burned-out sheds (Fig. 1). An explanation for the results from the 2 sheds being so similar (Table S4) could be that the 2 combustion trials were performed under similar combustion conditions with similar PAH formation and nature of particulate matter.

Although secondary emissions of PAH have been studied previously, the underlying mechanism has not been investigated. Results from this study show that secondary emissions from burned-out material can be significant. The following hypothesis for the appearance of the obtained results is presented, based on the equilibrium criterion of fugacity as the variable that governs the fate of a chemical in the environment (Mackay and Diamond 1989; Diamond and Mackay 1992). In order to investigate if equilibrium (steady state) was established between the PAH levels in air and PAHs on surfaces (walls, ceilings, burned-out material, etc.) at the end of the measurement period, the concentrations for each period were divided by those of period 7 for each PAH subgroup (Fig. S4). Air levels of H-PAH declined during a few days after the fire (period 3), after which the air levels were almost constant during the rest of the measurement period. Air levels of M-PAH were close to an equilibrium after about one month, while air levels of L-PAH did not reach equilibrium within the 38 d. Since the indoor environment was in natural contact with the outdoor environment via the window shutters, outdoor levels of PAH probably also affected the equilibrium process indoors. The octanol-air partition coefficient (K_OA_) has been suggested as a descriptor of the air-particle partitioning process (Finizio et al. 1997; Harner et al. 2000; Shoeib and Harner 2002; Odabasi et al. 2006). Compounds with log K_OA_ less than 8 or greater than 11 can take a very long time to reach equilibrium (Li et al. 2019), which agrees well with the results presented here.

Although the partitioning between the air and organic phase is sensitive to the K_OA_, it is negatively correlated with ambient temperature (Harner and Bidleman 1998; Odabasi et al. 2006; Huo et al. 2019), implying higher concentrations in the air at a higher temperature at equilibrium. Wind speed is another environmental parameter that may be important for the volatility of compounds and can give higher concentrations in the air (Li et al. 2019). The sheds were closed with low air flow and small temperature variations during the measurement period. A tendency for the levels to increase (all included PAH subgroups and DBT) during period 4, after 10 to 16 d, was observed in both sheds. The sheds were opened on 1 to 2 occasions during the measurement campaign for forensic investigations for educational purposes. It is possible that this happened during that period; however, this could not be confirmed. This observation, ie that air levels of both gaseous and particulate compounds could increase due to higher air movements, might be important from an occupational exposure point of view. Thus, the properties of a compound and the environmental parameters are of importance in the equilibrium process but also the nature of the particles (Finizio et al. 1997). For example, it has been reported that there may be a strong association between PAHs and soot particles (Odabasi et al. 2006). Neither the importance of temperature nor the nature of the particles for the equilibrium process was tested here.

Results were compared with studies relating to the working environment of firefighters in which secondary emissions of PAHs in non-fire situations have been suggested to contribute to the total exposure. In the study by (Sjöström et al. 2019), the GM levels of 16 US EPA PAHs for forensic investigators entering real fire sites within 12 h to 5 d after the fire was 3,500 ng m^−3^. This is comparable to the levels in the sheds in the present study after 2 wk or to the highest level measured for the post-fire workers in part 1 of the study (3,500 ng m^−3^). Elevated levels have been reported at 2 Polish fire stations where the mean levels in the common room, changing room and truck bay ranged between 1,800 and 6,000 ng m^−3^ (Rogula-Kozlowska et al. 2020). These levels are also comparable to the levels in the sheds after 2 wk. The prolonged outgassing of PAHs over days to weeks from indoor surfaces observed after an indoor fire are consistent with studies of semi volatile organic compounds in environmental tobacco smoke (third hand smoke) as well as volatile organic compounds in indoor air after major wildfire events (Jacob et al. 2017; Dresser et al. 2025).

The levels of the 16 US EPA PAHs during the 38-d measurement period were compared to those reported for the 16 US EPA PAHs in urban, rural, and indoor air in Sweden. Investigation of the impact of domestic wood burning on indoor PAH air levels reported the level of 27 gaseous and particulate PAHs to be 28 ng m^−3^ (Gustafson et al. 2008). A study of PAHs in an urban environment reported an indoor median concentration of 37 ng m^−3^ (Bohlin et al. 2008). The air levels in the sheds after 38 d were thus more than 15 times higher than ambient indoor air levels. However, these levels were comparable to the highest measured level of 16 US EPA PAHs (620 ng m^−3^) in a Swedish building with creosote impregnated construction material (Loive et al. 2024).

The BaP_eq_ level can be used to estimate risks in different occupational groups working in fire extinguishing or in post-fire tasks. It is important to emphasize that this calculation was intended to provide a measure of the toxicity of the PAH exposure and should therefore only be considered as guidance since the exposure during work tasks is far from lifelong and personal protective equipment is used. The BaP_eq_ levels were high in most of the personal measurements (part 1), mainly for the firefighters (Table 4). The samplers worn by the firefighters were mounted on the outside of the clothing and outside of their SCBA. Thus, this does not represent the inhalation exposure of the firefighters when extinguishing fires but represents an average value of the PAH toxicity outside the SCBA during the entire work shift.

In the post-fire study (part 2), the BaP_eq_ value was high the day after the fire (>20 ng m^−3^ BaP_eq_) but leveled off to 2 ng m^−3^ after 38 d (Fig. 2). After 12 h to 5 d, the standard time within which forensic technicians investigate a crime scene, the BaP_eq_ value had decreased to 13 ng m^−3^, still one order of magnitude higher than the annual standard of 1 ng m^−3^ BaP_eq_. This annual standard has been exceeded in urban sites all over the world, eg Zabrze, Poland (7.9 ng m^−3^ BaP_eq_) (Cwiklak et al. 2009), and Mexico City (1.3 ng m^−3^ BaP_eq_) (Mugica et al. 2010). Thus, after 38 d the indoor levels correspond to a polluted urban environment.

Calculations of PAH exposure toxicity using BaP_eq_ in work environments are sparse. Similar or higher BaP_eq_ values compared to those for firefighters in the present study were measured in a live fire training scenario, during fire extinguishing in Australia (4,400 to 63,000 ng m^−3^) (Kirk and Logan 2015). Two studies that assessed occupational exposure in non-fire work environments in fire stations in Portugal and Poland respectively, reported BaP_eq_ of 3 to 10 ng m^−3^ (Oliveira et al. 2017; Rogula-Kozlowska et al. 2020), which is comparable to the results for observers and post-fire workers.

Contribution of the toxicity of BaP alone of a PAH mixture is commonly more than 50% (Mugica et al. 2010; Jorgensen et al. 2013), and this was observed for firefighters in this study as BaP contributed 50 to 80% of the toxicity. The 2 ringed naphthalene (L-PAH) was the second largest with fractions between 4 and 20%. The 4-ringed fluoranthene (M-PAH), with a significant portion in the vapor phase ranked third, around 5%. Thus, the 2 PAHs that have a Swedish occupational exposure limit (OEL) (BaP; 2 µg m^−3^ and naphthalene 50 mg m^−3^) contributed the most to the toxicity of the exposure for this occupational group. No personal measurements of air levels of naphthalene exceeded the OEL, however, for BaP, the OEL was exceeded in personal measurements for 2 of the firefighters and measurements for an additional 2 firefighters exceeded 50% of the OEL. However, naphthalene is recognized as a relevant indoor pollutant by WHO which has recommended an annual guideline value in indoor air of 10 µg m^−3^ (WHO 2010). This value, which is a recommended guideline for the general population, was exceeded in 11 of the 13 firefighters and during the first measurement period of the second part of the study.

Gas phase PAHs, mainly naphthalene followed by fluoranthene and phenanthrene, accounted for over 70% of the total toxicity for 4 of the post-fire workers (part 1). These results are in line with work by Kander et al. showing that volatile PAH such as naphthalene readily penetrates traditional turn out gear (Kander et al. 2024, 2025). Thus, gas phase constituents can be significant to the total toxicity of PAH exposure for this occupational group. In contrast, for 2 post-fire workers BaP was dominant for the total PAH toxicity at approximately 80%, indicating a probable exposure route through particulate PAH when soot and dust is resuspended to air through mechanical agitation during post-fire work.

Contributions to the total toxicity of PAH exposure of individual compounds in the second part of the study (part 2) differed compared to the first part of the study. BaP constituted less than 1% of the toxicity throughout the measurement period in the post-fire study. L-PAHs and M-PAHs accounted for more than 99% of the total toxicity. From the beginning, the total toxicity was dominated by naphthalene (L-PAH) (30 to 50%) and fluoranthene (M-PAH) (20 to 30%). The L-PAHs acenaphthylene, fluorene, phenanthrene, and anthracene accounted for approximately 10% of the toxicity, respectively. The contribution to toxicity of naphthalene decreased over time and constituted <10% of the total toxicity after 38 d. Meanwhile, the contribution of fluoranthene to the total toxicity increased to 60 and 70% in the 2 sheds, respectively.

The estimated toxicities of PAH exposures presented here are based solely on the PAH with TEF values assigned, ie the 16 US EPA PAHs. Alkylated PAHs, detected at levels up to 20% of the total PAH content, are often more toxic than the parent PAH (Harner et al. 2013) and could contribute significantly to the exposure toxicity. It has further been shown that PAH derivatives such as nitrated and oxygenated PAHs which have been detected in the general environment (Albinet et al. 2007; Strandberg et al. 2023; Loive et al. 2024) are more toxic compared to parent PAHs (Abbas et al. 2018). These derivatives should also be included in studies of occupational exposure to PAHs. This will be the focus of future work.

## Strengths and limitations of study

The PUF-PAS sampler could be used in the extreme environment with elevated or high temperatures during extinguishing work. However, is not clear how adsorption to the sampler is affected by elevated temperatures, air turbulence, or moisture during fire extinguishing. Therefore, the results from the first part of the study should be regarded as quantitative estimations, especially for the firefighters.

Examples of variables which can affect the PUF-PAS performance in extreme firefighting conditions include concentrations of target compounds as well as environmental parameters such as temperature and wind speed (Cao and Hewitt 1991; Tuduri et al. 2006; Strandberg et al. 2018). Interfering compounds such as water and water vapor, as well as the size of particles to which the PAHs are associated can also affect uptake in PUF-PAS (Klánová et al. 2008; Harner et al. 2013; Bohlin et al. 2014a, 2014b; Melymuk et al. 2014; Wania and Shunthirasingham 2020). The density of the PUF material and the construction of the sampler housing may also affect the results. A further discussion of the accuracy of PUF-PAS for estimating air concentrations in firefighting conditions can be found in the SI. However, there were good agreements between duplicate measurements throughout the study; deviations were about 5 to 35% from the respective means for both gaseous and particulate PAHs (Fig. S1, Fig. S2). There was a slightly higher variation for some H-PAHs in the duplicate sample showing the lowest levels of PAHs, which is likely due to a greater uncertainty in the determination as the levels of H-PAHs were close to the LOD. A factor of 30 in concentration difference was found between the highest and lowest concentration in testing the concentration range of PUF-PAS for observers and post-fire workers, showing that the PUF-PAS can be used for reproducible measurements in this concentration range (Fig. S1).

In the calculations of toxicity of PAH exposure, only air exposure was considered. However, PAH exposure can occur either via inhalation, skin absorption, or orally, via hand to mouth contamination (Wingfors et al. 2018). It is important to understand the importance of both primary and secondary sources of exposure and the importance of absorption routes to reduce occupational exposure and risks. Secondary exposure can occur via evaporation of PAHs into the air from deposits on surfaces or via resuspension of larger fire particles from a fire source after the fire.

## Conclusions

A wide range of PAH exposures and exposure patterns were demonstrated for firefighters and post-fire workers. As differences were large both between the occupational groups and within each group, occupational exposure estimations are difficult to perform in these professions. The results also show that burns constituted sources for emission of PAHs, alkylated PAHs, and DBT. Thus, alkylated PAHs should be included when performing studies of exposure to PAH.

The second part of the study highlights that PAHs formed in the fire process are retained in indoor air and on indoor surfaces, leading to high concentrations in poorly ventilated areas for up to a week after a fire. Four weeks after burn, the levels of PAH and derivatives were still elevated compared to ambient air.

The PUF-PAS sampler functioned well under the extreme conditions during fire extinguishing exercises. However, the PAH air levels for the firefighters measured in the first part of the study should be considered as quantitative estimations.

Together, the results show that exposure at fire sites can pose significant health risks not only during the extinguishing work but also during subsequent work with materials and equipment, as well as investigating or cleaning the fire source days to weeks after the fire has been extinguished. The results may also be important for a general population perspective in fire scenarios such as forest fires.

## Recommendations

In general, fire scene investigations are performed within 5 d of the fire. Results suggest that PAH air exposure could be lowered by waiting at least 1 wk, preferably 2, before entering the fire scene. It is further important to avoid stirring up settled dust and particles which can lead to high exposure to the larger more carcinogenic PAHs. In situations where cleaning of the fire scene cannot wait, measures to reduce stirring up settled dust and particles, such as watering, could be considered.

The use of disposable nitrile gloves and P3 filter masks are important in situations that may involve secondary exposure, especially to compounds bound to particles. As many volatile fire smoke pollutants have been shown to penetrate protective clothing and coveralls (Kander et al. 2024, 2025), resulting in skin exposure, changing clothes and showering as soon as possible after a work shift is important.

## Supplementary Material

wxag003_Supplementary_Data

## Data Availability

Data are available on request.
