# Supplementary material for: PAH exposure and associated health risks can be high at a fire site both during fire and long after the fire is extinguished
Source: Ann Work Expo Health. 2026 Feb 4;70(2):wxag003. doi: 10.1093/annweh/wxag003 (PMC13017140; doi:10.1093/annweh/wxag003)
Supplement: wxag003_Supplementary_Data [file wxag003_supplementary_data.pdf]

Supporting information

PAH exposure and associated health risks can be high at a fire site both during fire and long after the fire is extinguished

Bo Strandberg[1,2], Karin Lovén[1,2], Vilhelm Malmborg[3], Jennie Özdemir[2], Joakim Pagels[3], Maria Hedmer[1,2] and Lina Hagvall[1,2*]

[1]Division of Occupational and Environmental Medicine, Department of Laboratory Medicine, Lund University, Lund, SE-221 00 Sweden

[2]Department of Occupational and Environmental Medicine, Skåne University Hospital, SE-22381 Lund, Sweden

[3]Division of Ergonomics and Aerosol Technology, Department of Design Sciences, Lund University, LTH, SE-22100 Lund, Sweden

*Corresponding author; Division of Occupational and Environmental Medicine, Lund University, Lund, Sweden

E-mail address: lina.hagvall@med.lu.se

Chemical analysis

All adsorbents, silica gel 60 (Merck, Darmstadt, Germany) and sodium sulfate (Merck, Darmstadt, Germany) were cleaned by thermal treatment at 450°C and activated at 100°C before use. All solvents were of glass distilled quality (Merck, Darmstadt, Germany). A deuterated internal standard mixture (1 ng μL^{-1}) containing the 16 US-EPA PAHs (Dr. Ehrenstorfer (Augsburg, Germany) were used. A native mixture at 1 ng μL^{-1}, containing 16 US EPA PAHs, benzo[e]pyrene, perylene and coronene (Dr. Ehrenstorfer, Augsburg, Germany), 15 alkylated and six DBT species (Ultra Scientific, North Kingstown, RI, USA), were used for detection and quantification of target compounds. Octachlorornaphthalene (OCN) (Ultra Scientific, North Kingstown, RI, USA) (1 ng μL^{-1}) was used as recovery determination standard (RDS). Quality controls (QCs) (SRM 1649a and 1649b) were purchased from the US National Institute of Standards and Technology (NIST) (Gaithersburg, MD, USA).

A Dionex ASE 350 Accelerated Solvent Extractor (Thermo Fisher Scientific, Inc. MA, USA) was used for extraction of the PUF-PAS samples. The samples were placed in extraction cells of 60 mL, containing 4 g of silica, spiked with 40 µL of the IS mixture and extracted using dichloromethane. The extraction was performed at 100°C and three static time cycles 5 min each. Following extraction, all samples were evaporated and transferred to 10 mL amber vials.

Sample extracts were concentrated by purging with nitrogen to a final volume of ca 0.5 mL. Extracts were then cleaned up through Pasteur pipettes filled with 2 cm of silica gel and some sodium sulphate on top, solvent exchanged to n-hexane and then concentrated by purging with nitrogen to a final volume of 40-70 µL.

Target compounds were separated on an Agilent 8890 GC System gas chromatograph coupled to an Agilent 7010B GC/TQ triple mass spectrometer (MS). Samples (0.2 µL) were injected using an Agilent 7693A autosampler unit. The capillary column used was a DB-5MS (30 m × 0.25 mm, 0.25 µm, Agilent Technologies). Helium was the carrier gas at a flow rate of 1.0 mL min^{-1}. The temperature program was as follows: initial temperature 50°C for 3 min; ramp at 10°C min^{-1} to 180°C and held for 5 min;

ramp at 3°C min^{-1} to 300°C and held for 20 min; injection at a transfer line temperature of 270°C. Electron impact ionization (EI) was performed for all target compounds at 70 eV energy and at a 280°C ion source temperature. The MS was operated in multiple reaction monitoring mode (MRM).

Quality control (QC) sample results

Two certified reference material (NIST SRM 1649a and 1649b urban dust) were used as quality control (QC) samples. Two QC samples of different SRM mass levels respectively (SRM 1649a QC1: 0.7 mg and QC2: 2.0 mg and SRM 1649b QC1: 0.5 mg and QC2: 3.0 mg) were applied and analyzed in parallel with the samples. The QCs were weighed on Teflon filters (Teflo: Pall Corporation, Port Washington, N.Y.) on a lab balance, submerged in 10 mL amber vials and extracted in 5 mL dichloromethane on a Sonica ultrasonic extractor (Soltec, Milan, Italy). The eluates were then purified in the same way as the other samples in the study (Pasteur pipettes filled with 2 cm of activated silica and sodium sulfate on top). The measured levels of 14 PAHs (SRM 1649a) and 21 PAHs (SRM 1649b) are for most part, within 30% of the certified levels (Figure S3). A slightly higher deviation was observed for the two most volatile PAHs in SRM 1649a. We assumed that slightly higher evaporation over time has occurred for these PAHs since the SRM material was acquired. We judged QC results to be acceptable.

Consideration and accuracy of PUF-PAS for estimating air concentrations in firefighter situations

The validity and reliability of a PUF-PAS as a quantitative tool for both gas and particle associated PAHs in short-term occupational measurements may be influenced by several variables. Variables affecting the PUF-PAS sampling rates of gaseous phase compounds include concentration of target compounds as well as environmental parameters such as temperature and wind speed. The variations in the concentration gradient around the sampler and the influence of interfering compounds can be significant for all three occupational groups in this study and especially for the

firefighters. Reportedly, a concentration difference of 20 affects uptake very little (Strandberg, Julander et al. 2018). The concentration during a measurement period, for all three occupational groups and especially for the firefighters, may have varied significantly more, which increases the uncertainty in personal exposure concentration determination. It is important to investigate how PUF-PAS responds to large concentration differences, during a measurement period both for PAH in gas and particle phase, to elevate the method as a quantitative tool. This will be the focus of future work. Moreover, both temperature and air turbulence can affect uptake of gaseous phase compounds in PUF-PAS. Air turbulence in a fire scenario is probably elevated, which can affect uptake in PUF-PAS. The uptake suggests being an air-side controlled mechanism and, thus, a higher wind speed lessens the boundary layer surrounding a passive sampler which could mean an increased uptake in PUF-PAS (Tuduri, Harner et al. 2006). In contrast, an elevated temperature which affects the compound-specific molecular diffusion coefficient would mean a lower uptake in PUF-PAS. An increase of 20°C will increase the diffusion by 4% (Cao and Hewitt 1991). The temperature during the fire extinguishing exercises was not measured in the study. Reportedly, the temperature during firefighting can be very high >180°C (Wingfors, Nyholm et al. 2018). How such high temperatures affect uptake in PUF-PAS is not known and need to be investigated further. Interfering compounds when it comes to firefighters during an extinguishing operation can be both other substances that are formed or released during the combustion but also water and water vapor. How the adsorption of PAH is affected by a moistened PUF-PAS also needs to be further investigated. It cannot be excluded that the samplers of some firefighters and observers may have been affected by moisture.

Sampling rates of particulate PAHs are contradictory in the literature. Some studies point to the same uptake for gaseous-phase as particulate PAHs (e.g., (Harner, Su et al. 2013, Strandberg, Julander et al. 2018). In contrast, several studies have shown that particle-bound fraction has a considerably lower uptake, which is ca 10% of that associated with the gaseous phase PAHs (e.g., (Klánová, Eupr et al. 2008)). The reason for such varying results is unclear, but may stem from the different conditions

(e.g., amount, size, and type of particles) associated with the various studies. The density of the PUFs and the sampler housing may also lead to variation in the results (Harner, Su et al. 2013, Bohlin, Audy et al. 2014, Bohlin, Audy et al. 2014, Melymuk, Bohlin et al. 2014, Wania and Shunthirasingham 2020). Often most of the PAHs are associated with the finest particles (1.5 µm), which are expected to diffuse in a similar manner to gaseous compounds. The accumulation (via diffusion) level of these particles in the PUF is similar to that of gaseous compounds (Melymuk, Bohlin et al. 2014). This implies that a fire (fire site) mainly generates fine particles and/or a distribution of PAHs around these particles. We believe the increased wind speed, the low PUF density used in this study and the open design used for personal measurements of this PUF-PAS application very well may have favored the uptake of particle-bound PAHs (Strandberg, Julander et al. 2018). We speculate that variation in concentration for particle-bound PAHs with the sampling design we use affects uptake in PUF-PAS only slightly, because the uptake of particles depends on how particles stick to the sampler via diffusion, unlike for PAHs in gaseous phase where uptake is driven by an equilibrium process. However, this is an important point to investigate further.

Thus, the variables concentration of target compounds as well as environmental parameters such as temperature and wind speed, can affect the absorption of PAH gas and particle phase completely differently. Therefore, it is important to emphasize that the uncertainty when using PUF-PAS may be higher for the firefighter group than the other two groups studied. It is therefore important to point out that the PAH results, especially for firefighters, should be considered as quantitative estimates. We tried to minimize the impact of temperature and wind speed by placing the PUF-PAS on the back of the helmet of this occupational group. Again, the effect of uptake of PAHs on a moistened PUF-PAS may have influenced some measurements on both firefighters as well observers.

We believe that the variables discussed above only slightly have affected the post-fire study (part 2) inside the burned-out sheds since the air flow was low. Thus, the temperature was assumed to be quite the same during the measurement period both day and night.

Table S1. Air concentrations (ng m^{-3}) of four individual PAHs and Dibenzothiophene, the sum of different PAH categories (L-, M-, and H-PAHs, alkylated-PAHs and 16 US EPA PAHs) and total PAHs, inside two burnt-out sheds. The results of the measurement periods refer to the average level for the days shown. Moreover, the background levels 200 m away are also given.

	Shed 1							Shed 2							
	Period 1	Period 2	Period 3	Period 4	Period 5	Period 6	Period 7	Period 1	Period 2	Period 3	Period 4	Period 5	Period 6	Period 7	Stationary[a]
Day	0--1	1--3	3--10	10--16	16--24	24--31	31--38	0--1	1--3	3--10	10--16	16--24	24--31	31--38	200 m away
Naphthalene	12000	3300	960	1100	520	460	210	11000	2500	780	1100	350	320	120	36
Phenanthrene	1300	1200	480	540	280	230	170	2500	1200	490	540	300	200	170	1.3
Fluoranthene	98	95	54	70	37	32	26	220	130	56	66	45	29	30	0.56
Benzo[a]pyrene	0.21	0.099	<0.010	<0.010	<0.010	<0.010	<0.010	0.29	0.23	0.010	0.011	<0.010	0.017	0.070	0.024
Dibenzothiophene	0.90	0.86	0.38	0.39	<0.32	<0.32	<0.32	1.8	0.84	0.42	0.35	<0.32	<0.32	<0.32	<0.32
L-PAHs	21000	9400	3400	3700	1900	1600	930	24000	7200	2900	3300	1500	1100	560	49
L-PAHs (excl. Naphthalene)	9800	6100	2400	2600	1400	1100	710	14000	4700	2100	2200	1100	780	450	14
M-PAHs	190	180	110	130	69	59	50	440	240	110	130	85	57	58	1.3
H-PAHs	2.6	1.4	0.52	0.45	0.33	0.28	0.23	4.0	2.7	0.43	0.34	0.30	0.45	0.83	1.5
16 US EPA PAHs	17000	6800	2400	2700	1400	1200	730	20000	5800	2200	2400	1200	860	480	44
Alkylated PAHs	4600	2800	1100	1200	620	450	240	5100	1700	870	9500	420	300	150	7.5
PAHs	22000	9600	3500	3800	2000	1600	980	25000	7400	3100	3400	1600	1200	620	52

[a]average concentration over 38 days performed with two measurement periods, ca 2.5 weeks each.

Table S2. Air concentrations (ng m^{-3}), geometric means (GMs) and ranges, determined via passive (PUF-cyl) sampling of individual, PAH categories (L-, M- and H-PAHs), 16 US EPA PAHs, alkylated PAHs, total PAHs and DBTs on firefighters and observers during controlled house burns and on post-fire workers with service and material maintenance after the fire, and two stationary measurement locations, 20 m and 200 m away, respectively.

	PAH category	Firefighters GM (n=13)	Range	Observers GM (n=4)	Range	Post fire workers GM (n=6)	Range	Stationary 20m away GM (n=3)	Range	Stationary 200 m away GM (n=3)	Range
naphthalene [a]	L-PAH	64000	4500-390000	3200	1500-8000	390	72-3000	6700	2700-13000	44	15-210
2-methylnaphthalene	L-PAH	6100	440-33000	8.6	<1.9-30	30	11-150	320	100-630	4.3	1.3-8.7
1-methylnaphthalene	L-PAH	4700	330-26000	12	<1.0-37	20	6.3-110	520	160-1100	4.6	0.80-13
biphenyl	L-PAH	5800	430-34000	27	6.8-170	16	4.3-85	1400	420-4300	16	9.9-24
2,3-dimethylnaphthalene	L-PAH	520	26-2700	7.3	4.7-22	2.4	0.74-8.0	49	16-88	0.96	0.28-2.1
acenaphthylene	L-PAH	13000	930-89000	250	59-1100	42	7.7-270	2000	590-5300	3.3	0.96-45
acenaphthene	L-PAH	1100	70-6700	23	6.6-78	4.8	1.2-25	160	46-370	1.6	0.79-4.8
2,3,5-trimethylnaphthalene	L-PAH	190	30-1000	3.9	2.2-8.2	3.1	1.9-5.8	13	7.3-18	1.3	<0.16-1.3
fluorene	L-PAH	3600	230-10000	66	18-270	12	2.7-65	630	180-1800	8.9	4.9-18
1-methylfluorene	L-PAH	290	14-2600	4.0	1.7-13	2.2	<0.65-3.0	16	4.1-44	2.5	<0.033-2.6
phenanthrene	L-PAH	6900	420-60000	97	23-460	16	3.6-66	1600	440-5600	17	8.1-47
anthracene	L-PAH	1200	64-12000	15	3.3-75	1.8	0.27-10	140	34-540	0.44	0.086-3.2
2-methylphenanthrene	L-PAH	260	9.3-1900	3.1	1.2-10	0.81	0.34-2.2	26	8.7-74	0.66	0.34-1.1
3-methylphenanthrene	L-PAH	230	6.8-1700	2.1	0.66-7.2	1.1	<0.44-1.5	29	11-72	0.90	0.43-1.6
1-methylphenanthrene	L-PAH	99	3.2-880	0.91	0.27-3.3	0.33	<0.092-0.49	9.4	2.8-28	0.21	0.13-0.43
1-methylanthracene	L-PAH	170	4.8-1300	2.0	0.86-4.8	0.67	<0.049-1.5	15	7.1-31	1.1	<0.030-2.7
2-phenylnaphthalene	L-PAH	330	12-4100	3.1	0.77-16	0.36	0.060-1.8	38	7.4-170	0.46	0.24-1.6
fluoranthene	M-PAH	630	26-6600	5.0	1.3-25	1.2	0.27-3.3	320	100-1200	8.4	5.0-18
pyrene	M-PAH	660	29-7700	5.0	1.3-25	1.4	0.22-4.0	270	82-920	5.4	<0.010-21
1-methylfluoranthene	M-PAH	180	1.9-3900	0.40	0.10-1.3	0.11	0.028-0.26	0.64	0.18-1.9	0.031	<0.010-0.031
retene	M-PAH	450	37-1900	4.6	2.9-6.7	3.1	<0.15-4.1	47	42-51	<0.010	<0.010
1-methylpyrene	M-PAH	100	3.2-1200	0.54	0.13-2.0	0.16	0.043-0.32	1.1	0.28-3.0	<0.010	<0.010
benzo(a)anthracene	H-PAH	610	24-8400	3.0	0.66-16	0.56	0.23-3.0	3.1	0.60-13	0.043	0.024-0.084
chrysene	H-PAH	1500	57-19000	7.7	1.9-41	1.6	0.54-8.4	15	2.7-68	0.44	0.38-0.57
2-methylchrysene	H-PAH	35	1.2-300	0.23	0.083-0.72	0.062	0.029-0.39	0.42	0.11-1.1	0.038	<0.030-0.038
5-methylchrysene	H-PAH	0.029	ND	0.029	<0.013-0.029	0.024	<0.013-0.025	0.046	<0.031-0.059	<0.031	<0.031
benzo(b)fluoranthene	H-PAH	210	10-2200	1.2	0.25-7.3	0.24	0.082-1.9	13	3.4-35	0.26	0.16-0.42
benzo(k)fluoranthene	H-PAH	140	7.3-1500	1.6	<0.21-5.8	1.4	<0.21-1.4	9.1	1.2-63	0.24	0.14-0.33
benzo(e)pyrene	H-PAH	120	6.7-1100	2.3	<0.35-4.4	1.3	<0.35-1.3	3.8	0.80-15	0.15	0.062-0.25
benzo(a)pyrene	H-PAH	540	33-5300	2.9	0.58-17	0.84	<0.35-4.1	11	2.0-47	0.15	0.10-0.19
perylene	H-PAH	160	8.2-1600	1.1	0.21-5.7	0.18	0.047-1.0	1.9	0.36-8.5	0.027	0.025-0.028
indeno(1,2,3-c,d)pyrene	H-PAH	240	15-2200	1.9	0.46-9.8	0.59	<0.20-3.3	15	3.2-59	0.66	0.40-0.91
dibenzo(a,h)anthracene	H-PAH	45	3.9-440	1.2	<0.20-1.8	0.49	<0.20-0.49	2.1	0.35-11	0.031	<0.010-0.031
benzo(g,h,i)perylene	H-PAH	280	19-2500	5.8	<0.86-10	4.2	<0.86-4.2	12	3.9-32	0.82	0.15-2.0
coronene	H-PAH	59	3.9-620	1.4	<0.33-2.6	0.84	<0.33-0.84	3.0	0.82-10	0.17	0.038-0.37
**L-PAHs**		90000	6300-580000	4100	1600-10000	480	91-3400	11000	4000-27000	79	30-330
**L-PAHs (excl. Naphthalene)**		26000	1800-190000	520	190-2000	77	16-450	4600	1300-14000	34	15-120
**M-PAHs**		1300	54-14000	11	2.6-50	2,6	0.49-6.6	590	180-2100	12	5-39
**H-PAHs**		3600	170-41000	21	3.9-110	3,6	0.78-27	82	17-330	2.9	1.6-4.3
**16 US EPA PAHs**		96000	6700-640000	3700	1600-10000	490	94-3500	12000	4200-29000	95	38-370
**Alkylated PAHs**		20000	15000-120000	76	21-330	76	33-380	2600	790-6600	34	18-59
**Total PAHs**		120000	8300-760000	3800	1600-11000	590	120-3600	15000	5000-36000	130	57-430
dibenzothiophene		21	1.0-250	0.91	<0.32-1.4	ND	<0.32	4.3	0.84-21	0.62	0.43-0.82
2-methyldibenzothiophene		4.2	<0.85-25	ND	<0.85	ND	<0.85	1.1	<0.85-1.1	<0.85	<0.85
1-methyldibenzothiophene		11	<0.61-85	ND	<0.61	ND	<0.61	1.8	0.70-5.3	2.4	<0.61-2.4
4-methyldibenzothiophene		2.3	0.082-19	0.11	<0.059-0.11	ND	<0.059	<0.059	<0.059	<0.059	<0.059
2,8-dimethyldibenzothiophene		0.43	0.067-3.5	0.050	<0.022-0.068	ND	<0.022	<0.022	<0.022	0.14	<0.022-0.16
2,4,7-trimethyldibenzothiophene		0.61	<0.091-5.2	ND	<0,091	ND	<0.091	<0.091	<0.091	0.14	<0.091-0.21
**Total DBTs**		32	1.3-390	0.25	0.058-1.5	ND	ND	6.4	1.5-27	1.2	0.69-3.4

[a]The 16 US EPA PAHs are in italic.

Table S3. Air concentrations (ng m^{-3}) of all individual PAHs, the sum of different PAH categories (L-, M-, and H-PAHs, alkylated-PAHs and 16 US EPA PAHs and DBTs) and a total of all 35 analyzed PAHs, the first and last period inside two burnt-out sheds. The results of the measurement periods refer to the average level for the days shown. Moreover, the background levels 200m away are also given.

	Shed 1		Shed 2		Stationary
	Period 1	Period 7	Period 1	Period 7	200 m away
Days	0--1	31--38	0--1	31--38	
naphthalene [a]	12000	210	11000	120	36
2-methylnaphthalene	840	26	850	15	2.5
1-methylnaphthalene	1500	50	1500	29	1.8
biphenyl	2100	140	2500	74	1.6
2,3-dimethylnaphthalene	100	9.3	140	7.8	0.24
acenaphthylene	2700	190	3800	48	3.2
acenaphthene	280	25	310	14	0.48
2,3,5-trimethylnaphthalene	16	2.1	21	1.8	0.31
fluorene	840	77	1600	56	1.2
1-methylfluorene	8.2	1.0	11	0.90	0.22
phenanthrene	1300	170	2500	170	1.3
anthracene	120	14	230	14	0.18
2-methylphenanthrene	15	2.9	22	2.8	0.081
3-methylphenanthrene	33	6.8	51	6.0	0.11
1-methylphenanthrene	13	3.1	20	2.9	0.033
1-methylanthracene	8.0	1.7	12	1.7	0.067
2-phenylnaphthalene	15	3.3	24	3.2	0.036
fluoranthene	98	26	220	30	0.56
pyrene	88	24	210	27	0.14
1-methylfluoranthene	<0.010	0.014	0.030	<0.010	0.011
retene	1.5	0.11	2.8	1.1	0.31
1-methylpyrene	0.13	<0.10	0.26	<0.10	0.016
benzo [a]anthracene	0.11	0.016	0.23	0.054	0.056
chrysene	0.15	<0.030	0.43	0.15	0.16
2-methylchrysene	<0.030	<0.030	<0.030	<0.030	0.062
5-methylchrysene	<0.030	<0.030	<0.030	<0.030	<0.013
benzo[b]fluoranthene	0.49	0.13	0.74	0.21	0.24
benzo[k]fluoranthene	0.32	0.042	0.65	0.24	0.14
benzo[e]pyrene	0.32	0.016	0.36	0.040	0.13
benzo[a]pyrene	0.21	<0.010	0.29	0.070	0.024
perylene	0.054	<0.020	0.079	<0.020	0.0080
indeno[1,2,3-c,d]pyrene	0.29	<0.010	0.47	0.10	0.49
dibenzo[a,h]anthracene	0.091	<0.010	0.045	<0.010	0.049
benzo[g,h,i]perylene	0.61	0.031	0.68	0.070	0.32
coronene	<0.010	<0.010	0.019	<0.010	0.084
**L-PAHs**	21000	990	24000	620	49
**L-PAHs (excl. Naphthalene)**	10000	780	14000	510	14
**M-PAHs**	190	51	440	58	1.3
**H-PAHs**	2.5	0.61	4.0	1.1	1.5
**16 US EPA PAHs**	17000	730	20000	480	44
**Alkylated PAHs**	4600	310	5100	210	7.5
**DBTs**	0.90	<0.32	1.8	<0.32	<0.32
**PAHs**	22000	1000	25000	680	52

[a]The 16 US EPA PAHs are in italic.

Table S4. Spearman's rank correlation coefficient (r_s) between Shed 1 and Shed 2 for air levels of a selection of PAHs, all 35 analysed PAH divided into L-, M-, and H-PAH as well as for dibenzothiophene, calculated using bivariate Spearman correlation analysis.

Compound	r_s	p-value
naphthalene	1.0	<0.01
acenaphthylene	1.0	<0.01
fluorene	1.0	<0.01
phenanthrene	0.96	<0.001
1-methyl-naphthalene	1.0	<0.01
3-methylphenanthrene	0.93	<0.01
2-phenylnaphthalene	0.96	<0.001
fluoranthene	0.96	<0.001
pyrene	0.96	<0.001
1-methylpyrene	1.0	<0.01
benzo[a]anthracene	0.54	NS
benzo[b]fluoranthene	0.79	0.036
benzo[e]pyrene	0.86	0.014
benzo[a]pyrene	0.80	0.03
benzo[g,h,i]perylene	0.75	NS
L-PAH (17 compounds)	1.0	<0.01
M-PAH (5 compounds)	0.96	<0.001
H-PAH (13 compounds)	0.86	0.014
dibenzothiophene	1.0	<0.01

Table S5. Individual toxic equivalents of benzo[a]pyrene (BaP_{eq}) for the 16 US EPA prioritized PAH.

	**Shed 1**							**Shed 2**						
**BaP_{eq} (ng m^{-3})**	Period 1	Period 2	Period 3	Period 4	Period 5	Period 6	Period 7	Period 1	Period 2	Period 3	Period 4	Period 5	Period 6	Period 7
naphthalene	12	3.3	0.96	1.1	0.52	0.46	0.21	11	2.5	0.78	1.1	0.35	0.32	0.12
acenaphthylene	2.7	1.1	0.41	0.48	0.31	0.27	0.19	3.8	0.94	0.34	0.45	0.24	0.16	0.048
acenaphthene	0.28	0.20	0.088	0.087	0.046	0.036	0.025	0.31	0.12	0.050	0.058	0.029	0.024	0.014
fluorene	0.84	0.63	0.32	0.27	0.14	0.11	0.077	1.6	0.62	0.28	0.22	0.12	0.082	0.056
phenanthrene	1.3	1.2	0.48	0.54	0.28	0.23	0.17	2.5	1.2	0.54	0.49	0.30	0.20	0.17
anthracene	1.2	1.1	0.54	0.52	0.24	0.19	0.14	2.3	1.2	0.61	0.45	0.26	0.18	0.14
fluoranthene	4.9	4.8	2.7	3.5	1.9	1.6	1.3	11	6.3	3.3	2.8	2.2	1.5	1.5
pyrene	0.088	0.079	0.047	0.058	0.030	0.026	0.024	0.21	0.11	0.061	0.053	0.040	0.027	0.027
benzo(a)anthracene	0.011	0.011	0.0056	0.0068	0.0023	0.0014	0.0016	0.023	0.018	0.0044	0.0037	0.0021	0.0023	0.00054
chrysene	0.0015	0	0.0009	0.0009	0	0	0	0.0043	0.0036	0.0005	0	0	0	0.0015
benzo(b)fluoranthene	0.049	0.027	0.0096	0.0060	0.0078	0.0077	0.013	0.074	0.043	0.0058	0.0070	0.011	0.012	0.021
benzo(k)fluoranthene	0.032	0.028	0.0056	0.0066	0.0074	0.0056	0.0042	0.064	0.038	0.0048	0.0047	0.0083	0.0090	0.014
benzo(a)pyrene	0.21	0.099	0	0	0	0	0	0.29	0.23	0.010	0.011	0	0.0017	0.070
indeno(1,2,3-c,d)pyrene	0.029	0.017	0.017	0.011	0.011	0.0083	0	0.047	0.042	0.013	0.011	0.0061	0.013	0.0098
dibenzo(a,h)anthracene	0.10	0	0	0	0	0	0	0.049	0.020	0.051	0	0	0	0
benzo(g,h,i)perylene	0.012	0.0051	0.0007	0.0007	0.0005	0.0007	0.0006	0.014	0.0072	0.0007	0.0008	0.0005	0.0010	0.0014
coronene	0	0	0.0001	0	0	0	0	0.0004	0.0004	0	0	0	0	0
Sum BaP_{eq}	23	13	5.6	6.6	3.5	2.9	2.2	33	13	6.1	5.6	3.6	2.5	2.2

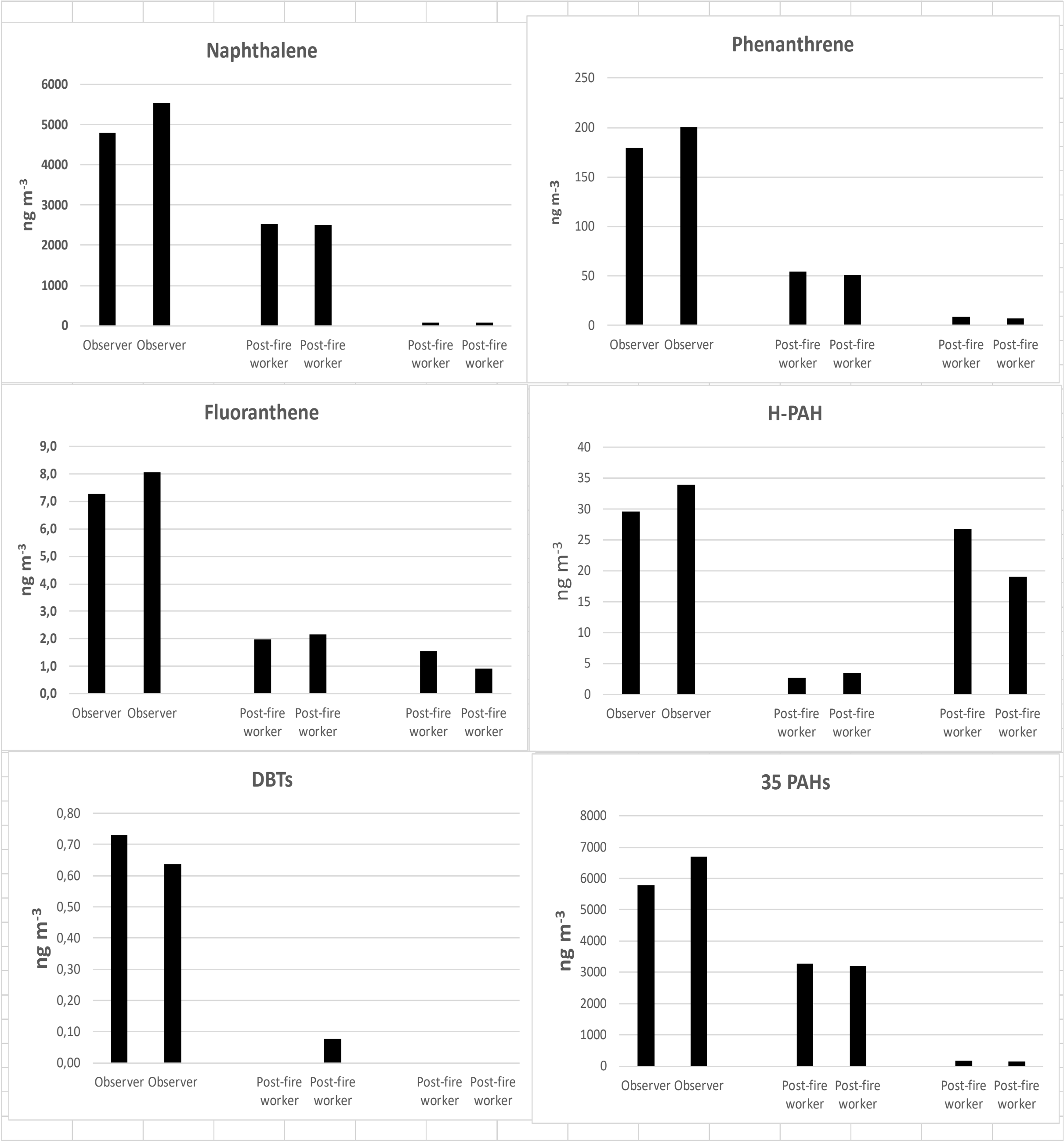

Figure S1. Duplicate sampling results (ng m^{-3}) in study part 1, two PUF-PAS placed on the same person, one observer and two post-fire workers of four individual components (naphthalene, phenanthrene, fluoranthene and total DBTs), high molecular weight PAHs (H-PAH) and sum PAHs.

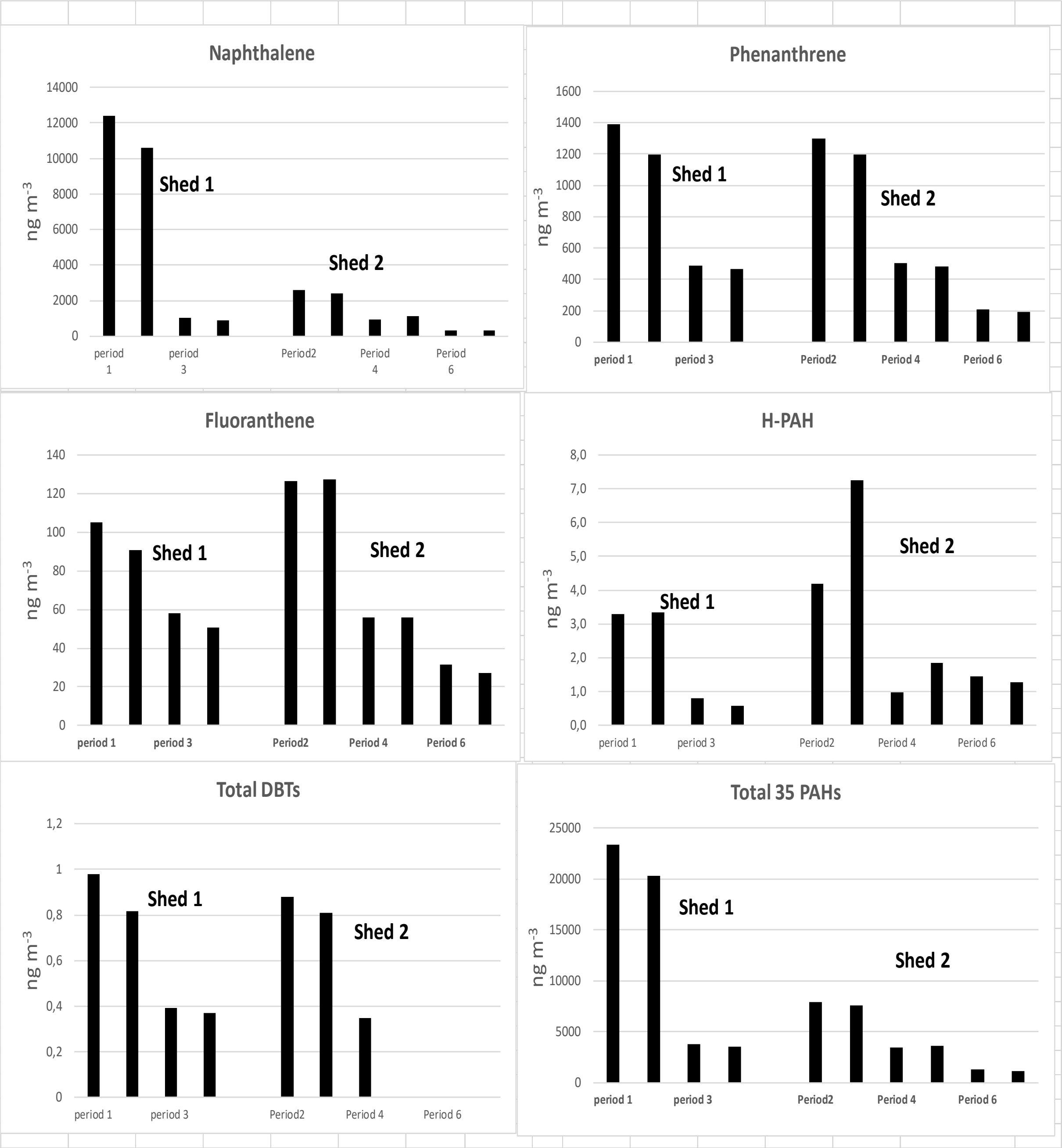

Figure S2. Duplicate sampling results (ng m^{-3}), two PUF-PAS placed next to each other for five of the seven measurement periods in the post fire study (part 2) of four individual components (naphthalene, phenanthrene, fluoranthene and dibenzothiophene), high molecular weight PAHs (H-PAH) and total 35 PAHs. The duplicate measurement alternated between the two sheds.

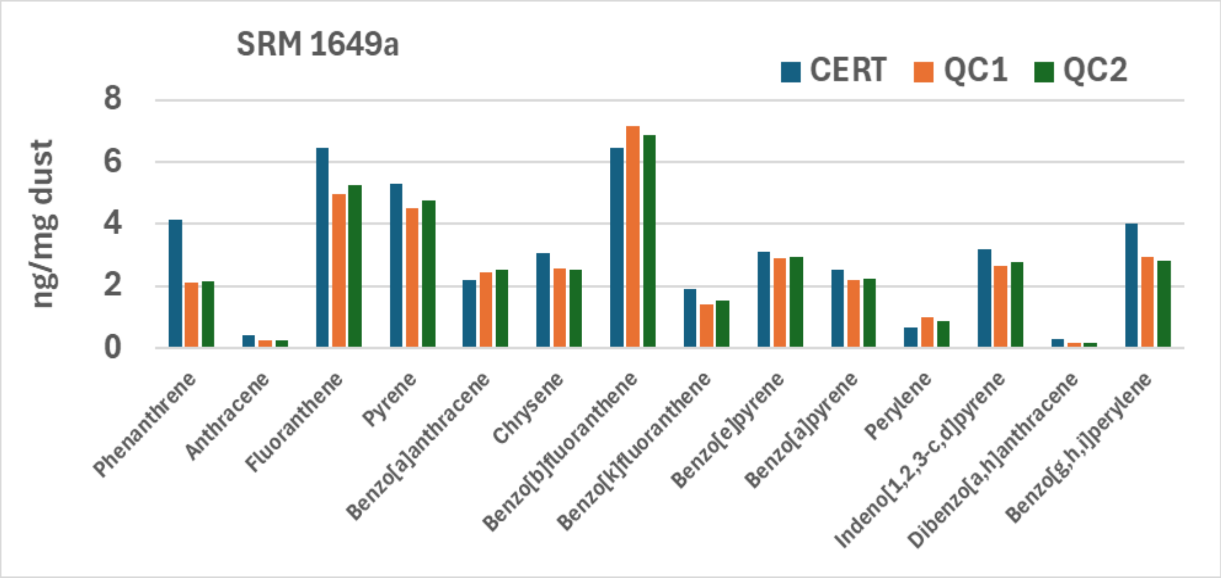

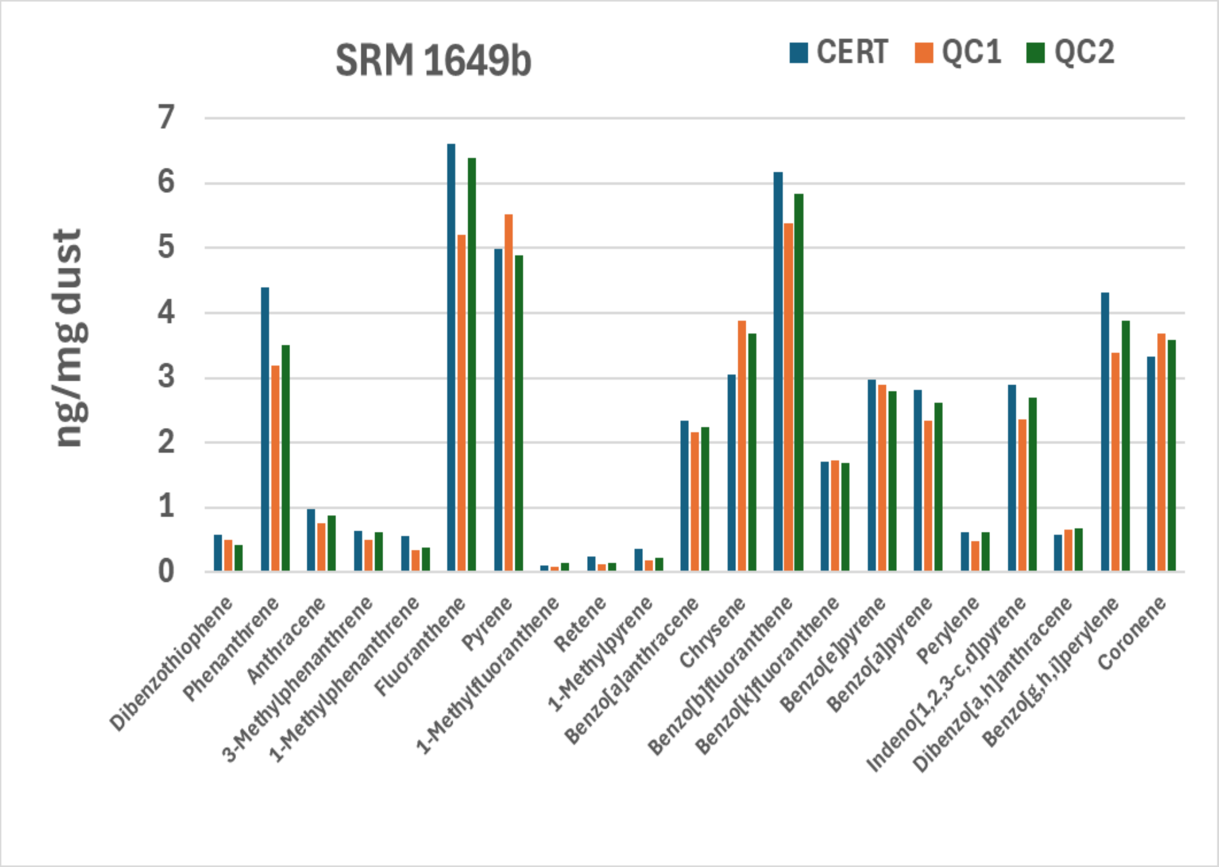

Figure S3. Results of two quality controls (QCs) samples (ng/mg dust) of two certified materials (NIST urban dust SRM 1649a and1649b), compared to published certified (CERT) values of 14 and 21 polycyclic aromatic compounds (PAHs), respectively.

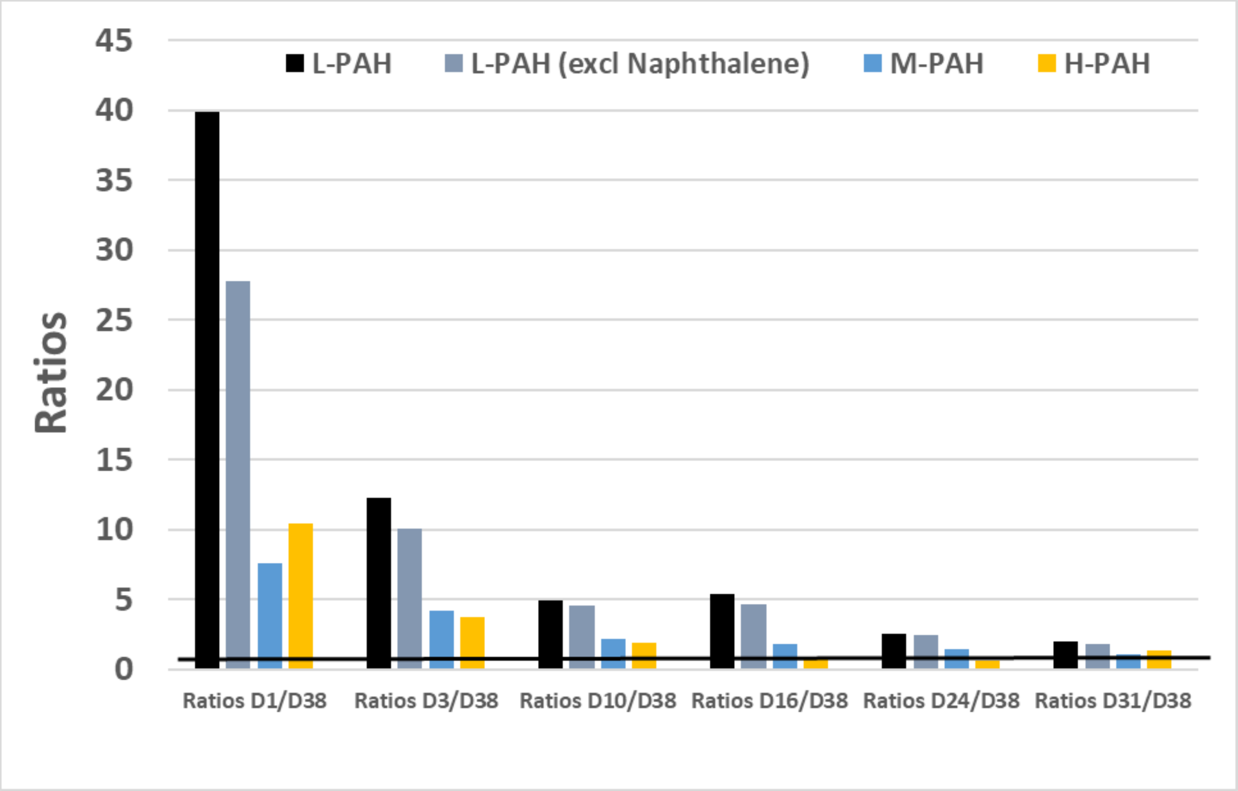

Figure S4. Concentration ratios between the measurement points (day 1, 3, 10, 16, 24 and 31) and the last (day 38) for the PAH subgroups L-PAH, L-PAH (exclusive naphthalene), M-PAH and H-PAH. The ratio around one (the bold line) is assumed to have established equilibrium.

References

Bohlin, P., O. Audy, L. Skrdlikova, P. Kukucka, P. Pribylova, R. Prokes, S. Vojta and J. Klanova (2014). "Outdoor passive air monitoring of semi volatile organic compounds (SVOCs): a critical evaluation of performance and limitations of polyurethane foam (PUF) disks." Environmental Science-Processes & Impacts **16**(3): 433-444.

Bohlin, P., O. Audy, L. Skrdlikova, P. Kukucka, S. Vojta, P. Pribylova, R. Prokes, P. Cupr and J. Klanova (2014). "Evaluation and guidelines for using polyurethane foam (PUF) passive air samplers in double-dome chambers to assess semi-volatile organic compounds (SVOCs) in non-industrial indoor environments." Environmental Science-Processes & Impacts **16**(11): 2617-2626.

Cao, X. L. and C. N. Hewitt (1991). "APPLICATION OF PASSIVE SAMPLERS TO THE MONITORING OF LOW CONCENTRATION ORGANIC VAPORS IN INDOOR AND AMBIENT AIR - A REVIEW." Environmental Technology **12**(11): 1055-1062.

Harner, T., K. Su, S. Genualdi, J. Karpowicz, L. Ahrens, C. Mihele, J. Schuster, J. P. Charland and J. Narayan (2013). "Calibration and application of PUF disk passive air samplers for tracking polycyclic aromatic compounds (PACs)." Atmospheric Environment **75**: 123-128.

Klánová, J., P. Eupr, J. Kohoutek and T. Harner (2008). "Assessing the influence of meteorological parameters on the performance of polyurethane foam-based passive air samplers." Environmental Science & Technology **42**(2): 550-555.

Melymuk, L., P. Bohlin, O. Sánka, K. Pozo and J. Klánová (2014). "Current Challenges in Air Sampling of Semivolatile Organic Contaminants: Sampling Artifacts and Their Influence on Data Comparability." Environmental Science & Technology **48**(24): 14077-14091.

Strandberg, B., A. Julander, M. Sjostrom, M. Lewne, H. K. Akdeva and C. Bigert (2018). "Evaluation of polyurethane foam passive air sampler (PUF) as a tool for occupational PAH measurements." Chemosphere **190**: 35-42.

Tuduri, L., T. Harner and H. Hung (2006). "Polyurethane foam (PUF) disks passive air samplers: Wind effect on sampling rates." Environmental Pollution **144**(2): 377-383.

Wania, F. and C. Shunthirasingham (2020). "Passive air sampling for semi-volatile organic chemicals." Environmental Science-Processes & Impacts **22**(10): 1925-2002.

Wingfors, H., J. R. Nyholm, R. Magnusson and C. H. Wijkmark (2018). "Impact of Fire Suit Ensembles on Firefighter PAH Exposures as Assessed by Skin Deposition and Urinary Biomarkers." Annals of Work Exposures and Health **62**(2): 221-231.
